# Composition of Corroded Reinforcing Steel Surface in Solutions Simulating the Electrolytic Environments in the Micropores of Concrete in the Propagation Period

**DOI:** 10.3390/ma15062216

**Published:** 2022-03-17

**Authors:** Pascual Saura, Emilio Zornoza, Carmen Andrade, Verónica Ferrandiz-Mas, Pedro Garcés

**Affiliations:** 1 Department of Architectonical Constructions, University of Alicante, San Vicente, 03690 Alicante, Spain; pascual.saura@ua.es; 2 Department of Civil Engineering, University of Alicante, San Vicente, 03690 Alicante, Spain; emilio.zornoza@ua.es; 3 International Center of Numerical Methods in Engineering (CIMNE), 28010 Madrid, Spain; candrade@cimne.upc.edu; 4 Centre for Innovative Construction Materials (CICM), University of Bath, Bath BA2 7AY, UK; vfm24@bath.ac.uk

**Keywords:** steel reinforced concrete, polarization, Raman, XPS

## Abstract

In the present work, the composition of a corroded reinforcing steel surface is studied at different pH values (related to different degrees of development in the corroding zones of the corrosion process) in solutions simulating chloride-contaminated environments. The media considered consist of saturated calcium hydroxide solutions, progressively neutralized with FeCl_2_ or by adding 0.5 M NaCl to the solution. The results found in present work confirm higher levels of acidity in the solutions with higher concentrations of Fe^2+^.In the present work, emphasis is given to the composition of the oxides in solutions that simulate the conditions that exist inside of a localized corrosion pit as a consequence of the reaction of chloride on reinforcing steel. The oxides were studied using Raman and XPS techniques; the results obtained with both techniques are mutually coherent. Thus, in the passive state, the oxides found are those reported previously by other authors, while in the corroding state, the present results are more comprehensive because the conditions tested studied a variety of pore solution composition with several pH values; we tried to reproduce these values inside the pits in conditions of heavy corrosion (very acidic). The oxides found are those typically produced during iron dissolution and seem not the best route to study the corrosion process of steel in concrete; the electrochemical tests better characterize the corrosion stage.

## 1. Introduction

It is accepted that reinforced concrete is the most suitable material to be used for structural purposes in civil engineering applications. Under normal service conditions, concrete has an alkaline pore solution (pH ≥ 12.6) [1] that guarantees the passivation of the steel reinforcements, in addition to being a physical barrier against the penetration of environmentally aggressive agents. However, several chemical processes can modify the passive state of steel reinforcements by means of a decrease in the pore solution’s pH value, leading to an increase in the steel corrosion rate [2,3]. The problems caused by the corrosion of the reinforcements usually lead to phenomena such as: a loss of adherence between the steel and concrete, a reduction in the steel section, and cracking in the concrete cover. The main strategy for avoiding the corrosion of steel in concrete should begin by preparing concrete with lower porosity, such as that with self-curing admixtures. However, when damage is detected, other actions can be implemented. For example, some authors propose to monitor the early corrosion evolution of the rebar and take early steps to overcome the situation [4].

One of the main causes of localized attack [2,3] to the steel reinforcement is the penetration of chloride ions through the porous reinforcement cover [5,6,7]. The critical chloride concentration needed to break the passive state is not constant, but it depends on several factors, such as the cation type of the chloride salt [8].

When a chloride-induced corrosion process occurs, corrosion pits develop on the surface of the steel reinforcement, so a local corrosion phenomenon takes place, unlike the generalized corrosion mechanism that is produced under carbonation-induced corrosion. This local corrosion process is especially harmful since a progressive acidification of the environment inside the pit takes place due to the increase in the Fe^2+^ concentration; therefore, the corrosion rate increases as a consequence of the low pH solution created inside the corrosion pit [9].

In previous research [10], the progressive acidification produced inside the corrosion pits during the evolution of a chloride-induced corrosion process was investigated. This situation is now extended to the identification of the corrosion products produced at the different pH values typical of the passive condition and in the steel–solution interface or inside the pits, where a high concentration of FeCl_2_ can exist. These conditions are to be reproduced in the tests by solutions of different chloride concentrations.

On the one hand, regarding the behavior of reinforcement bars rolled from other compositions, such as low-alloy steel having Cu and Cr, 2–3 times slower corrosion rates are exhibited than in plain carbon steel exposed in chloride-contaminated concrete pore solutions, as well under actual embedded conditions [11].

More recently [12], the mechanisms of the formation of passive films and corrosion pits for Cr-modified reinforcing steels in saturated Ca(OH)_2_ solutions with chlorides, by the addition of NaCl, were proposed. Improved pitting corrosion resistance was evident for steels with increasing Cr content, which was primarily due to the formation of Cr-enriched compact rust layers, as well as stable corrosion pits for Cr-modified steels.

The composition of the oxides generated during the development of the corrosion process of iron in alkaline solutions was previously investigated by other authors [13,14,15], who found a large variety of different ferrous and ferric oxides and hydroxihydroxydes depending on the testing solutions and conditions. The most used techniques in this type of work are X-ray diffraction analysis (XRD), infrared spectrophotometry (IR), XPS and Raman spectroscopy, and in relation to the electrochemical methods, voltammetry and polarization resistance to monitor the corrosion evolution are the most common.

Iron oxides and oxyhydroxides, which are the main products of the metallic corrosion process, absorb infrared radiation. This is the reason that such an important number of studies related to the composition of corrosion products use IR spectrophotometry. However, Raman spectroscopy offers at least two advantages over IR spectrophotometry: there is no need of a sample preparation and the spectra of the metal–water interface are quickly and easily obtained.

Another technique that can be used on metallic surfaces is X-ray photoelectronic spectroscopy (XPS) [16]. Thus, the elemental chemical composition of the metallic surface and the nature of the chemical bonding that exists between different elements can be identified.

The main objective of the present research is to study the potential correlation between the evolution in the corrosion degree of the chloride-induced corrosion process in the steel, simulated by increasing acidity in the solutions tested, and the chemical species of iron oxides that can be found in the corroding surface. That is, the present work emphasizes the characterization of the corrosion products throughout the evolution of the solution assumed to be inside the corrosion pit. In the tests, the corrosion of the steel bars is generated by the addition of FeCl_2_ and NaCl to alkaline solutions. The evolution of the corrosion is monitored through the polarization resistance technique. In addition, at certain stages, through the application of the Raman spectrometry and XPS techniques, the composition of the oxides is studied.

## 2. Experimental

### 2.1. Corrosion Rate Measurements

Corrugated reinforced steel bars were used for the experiments, the composition of which is given in Table 1. The exposed area of the reinforcing steel electrodes was 6.00 cm^2^. Prior to the experiment, the reinforced steel electrodes were cleaned in HCl:H_2_O 1:1 solutions with hexamethylenetetramine, abraded with abrasive paper and degreased in acetone. Adhesive tape was used for limiting the active area.

A series of solutions simulating the pore solution evolution during the corrosion initiation period were prepared [17,18,19]. Table 2 shows the solutions studied and their initial pH and conductivity values. The prepared solutions for the experiments were: a control solution of saturated Ca(OH)_2_, which was also prepared to compare with a reference system, NaCl 0.5 M, and FeCl_2_ 0.02 M, 0.2 M and 2 M. These solutions would simulate the progressive acidification that takes place during the corrosion propagation period due to the release of Fe^2+^ ions, which reacts with H_2_O to yield Fe(OH)_2_ and H^+^ according to Equation (1) [7,20]:

Fe^2+^ + 2 H_2_O → Fe(OH)_2_ ↓ + 2 H^+^ (1)

The corrosion cell was similar to that used in previous studies [17,18,19]. Three identical reinforcing bars and a carbon auxiliary electrode were immersed in each cell (3 steel electrodes in the same cell were tested at the same time). The corrosion rate results are always the average of the three reinforcing steels immersed in the cell.

The conductivity and the pH of the solutions were measured several times during and at the end of each test. A Crison model 2002 ion analyzer microprocessor pH meter and a combined electrode for the pH range 0–14 were used to measure pH values. Conductivity measurements were taken from a Crison model 525 conductometer.

The electrochemical technique used to measure the corrosion rate (*I_corr_*) was the polarization resistance method (*R_p_*) [21,22,23]. The *R_p_* is the slope of the polarization curve around the corrosion potential: *R_P_* = ΔE/ΔI when ΔE→0. The *R_P_* (kΩ·cm^2^) value is related to *I_corr_* (µA/cm^2^) by means of a constant, denominated *B* (mV/decade) by Stern and Geary [24].

(2) $$I_{corr}=\frac{B}{R_{p}}$$

The constant *B* is related to Tafel’s constants *β_a_* (mV/decade) and *β_c_* (mV/decade) by Equation (3):

(3) $$B=\frac{\left(\beta_{a}\cdot \beta_{c}\right)}{2.303\left(\beta_{a}+\beta_{c}\right)}$$

Stern and Geary [24] stated that when using a mean *B* value of 26 mV/decade, the maximum Icorr error factor is 2. For the case of steel embedded in concrete, a value of 13 mV/decade was found for the active state (corrosion), whereas 52 mV/decade is more appropriate for passive steel.

*R_p_* and corrosion potential were periodically measured during the experiment. In order to validate the electrochemical Icorr values obtained through *R_p_* measurements, they were compared by means of Faraday’s law to the gravimetric losses obtained in the same reinforcing steels.

Along with the results obtained, the mean Icorr (µA/cm^2^) was also used. The mean Icorr values were obtained by dividing the area of the Icorr plots (integration of the Icorr vs. time curves) by the duration of each test (expressed in days). The quantity mean Icorr represents the average Icorr value in the testing period.

### 2.2. Raman Spectroscopy Measurements

Raman spectra were obtained using the LabRam spectrometer model (Jobin-Yvon–Horiba company, Kyoto, Japan), equipped with a confocal microscope. A 200 nm aperture was used with a confocal opening of 600 nm. The excitation source was a 17 mW He-Ne laser at a 632.8 nm wavelength. The laser beam focused on the sample in a 2 µm spot through a 50× Olympus long-distance objective lens with a numeric aperture of 0.5. In all of the cases studied, the resolution of the spectrometer was better than 3 cm^−1^ using a charge-coupled device (CCD) detector with dimensions of 1064 × 256 pixels, cooled by the Peltier effect. The samples used in the analysis were obtained through 5 mm cylindrical cuts from corrugated steel bars. Each sample was previously introduced in study solutions. The Raman spectra were measured at 1, 5, and 90 days.

### 2.3. Measurements Made through X-ray Photoelectron Spectroscopy (XPS)

This analysis was carried out with a VG-Microtech Multilab electronic spectrometer (Thermo Scientific, Uckfield, UK). The samples were irradiated with X-rays emitted from a Mg anode (K_α_ 1253.6 eV emissions) using a constant energy analyzer with a 50 eV step. The X-ray emission potential was 300 W (20 mA at 15 kV). The pressure in the interior of the analysis chamber was maintained at 5·10–10 mbars at a temperature of 173 K. The scale of the bond energy (BE) was calibrated with reference to C 1 s to 284.6 eV. The BE measurement precision was ±0.2 eV. Depending on the scale of the utilized energy, the range of spectral resolution oscillated between 0.10 and 0.15 eV. The values of BE were obtained using the PeakFit program included with the spectrometer software.

## 3. Results

### 3.1. Corrosion Rate Tests

**Figure 1:**
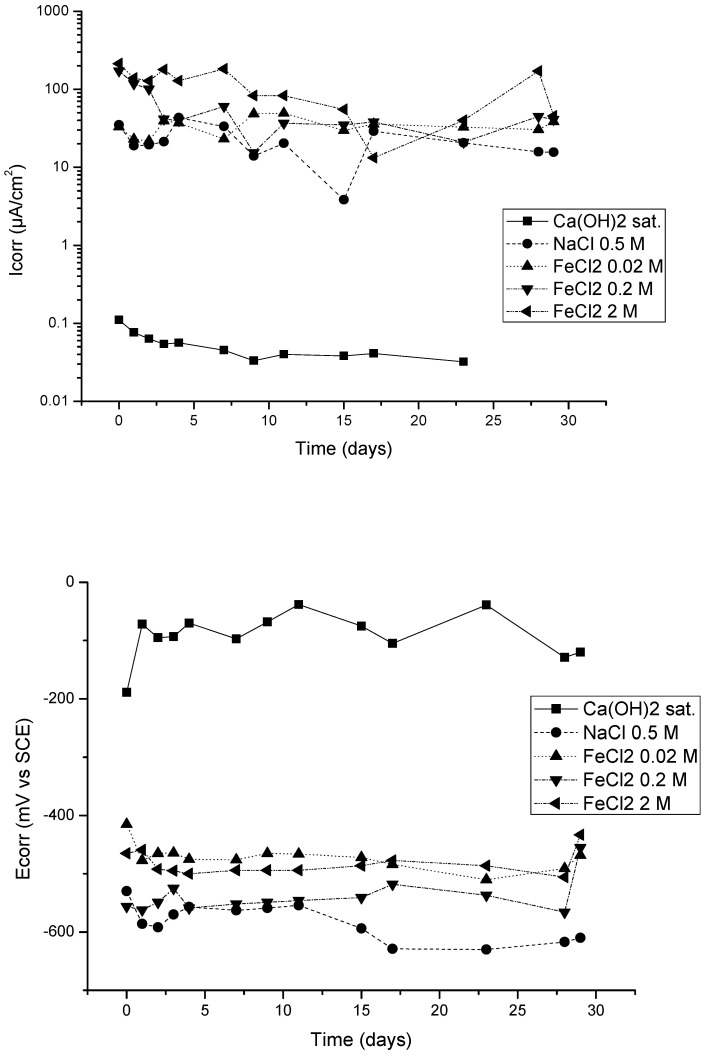
Upper part: the corrosion rate (µA/cm^2^); lower part: the corrosion potential (mV vs. SCE).

Figure 1 presents the evolution of the corrosion rate (upper figure) and of the corrosion potential (lower figure) with time. While the solution without added chlorides showed very low corrosion rates because the steel passivates, i.e., the other solutions, showed extremely high corrosion rates, such as that of 0.2 M of FeCl_2_.

**Figure 2:**
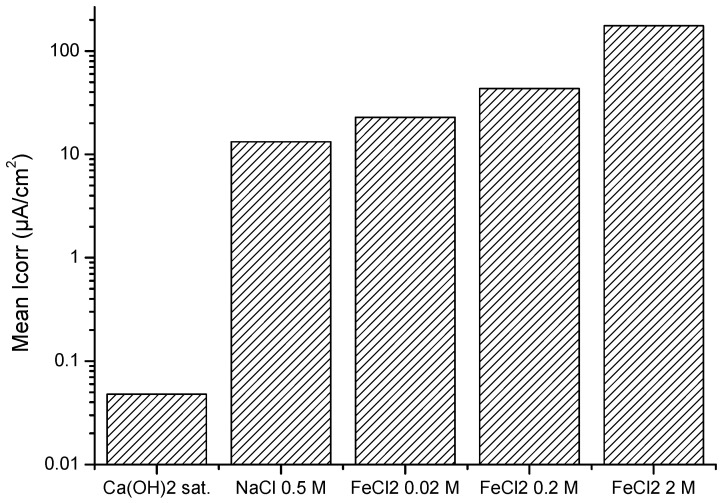
Mean Icorr values registered for the steel bars in the studied solutions.

Figure 2 shows the average corrosion rate across the 28 days of testing. Steel samples in more corrosive environments (i.e., environments with a higher concentration of chlorides and a lower pH as a consequence of the hydrolysis produced through a higher concentration of Fe^2+^) offered a higher corrosion rate. These results indicate the higher level of aggressiveness of the solutions with higher concentrations of Fe^2+^.

### 3.2. Raman Spectroscopy Analyses

**Figure 3:**
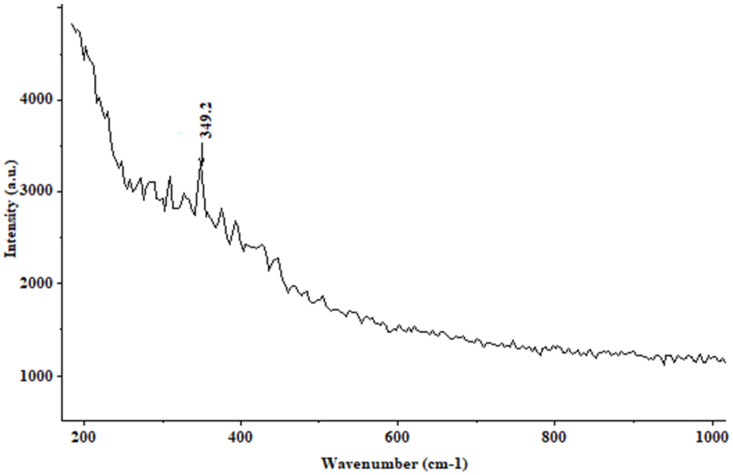
Raman spectrum corresponding to the reference steel sample in the air (steel).

Figure 3 shows the Raman spectrum corresponding to the reference steel sample in the air. Note the slight peak at 349 cm^−1^, indicating steel.

**Figure 4:**
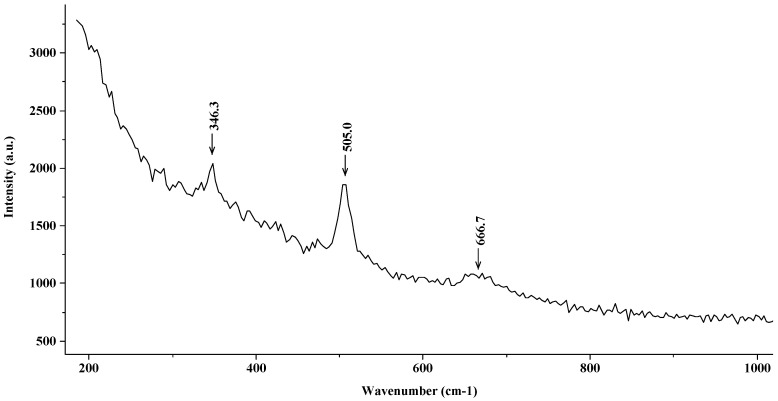
Raman spectrum (maghemite (γ-Fe_2_O_3_)) corresponding to the steel introduced into a saturated solution of Ca(OH)_2_.

Figure 4 shows the Raman spectrum corresponding to the steel introduced into a saturated solution of Ca(OH)_2_ over a four-day period. First of all, the characteristic peaks corresponding to maghemite (γ-Fe_2_O_3_) located at 505 cm^−1^ and 666 cm^−1^ are observed. This compound forms part of the passivating, continuous, and compact film, which is typically found in these media.

**Figure 5:**
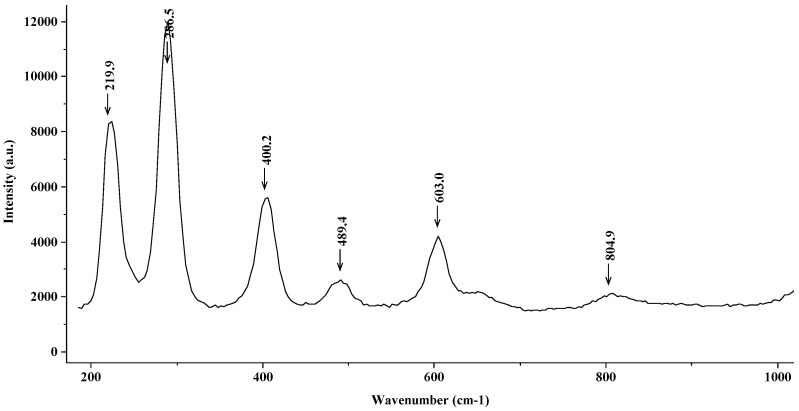
Raman spectrum (hematite (α-Fe_2_O_3_)) corresponding to the steel introduced into a 0.5 M solution of NaCl.

Figure 5 shows the Raman spectrum corresponding to the steel in a NaCl 0.5 M solution. The signal observed in the spectrum corresponds to hematite (α-Fe_2_O_3_), with characteristic peaks situated at 225 cm^−1^, 299 cm^−1^, 412 cm^−1^, 500 cm^−1^, and 613 cm^−1^, which are observed although slightly displaced in the spectrum in Figure 5. These small differences, as concluded by Faria [25], may be due to the use of lasers beams with different potential. This medium could bring about a change from hematite to the formation of lepidocrocite (γ-FeOOH), as established by Thierry [16] and Nauer [26].

**Figure 6:**
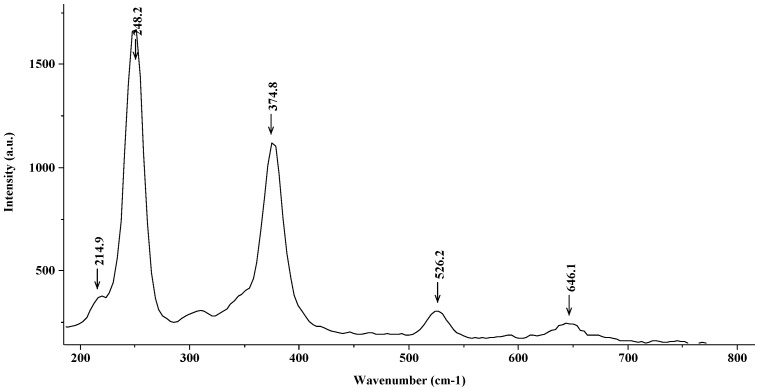
Raman spectrum (lepidocrocite (γ-FeOOH)) corresponding to the steel introduced into a 0.02 M solution of FeCl_2._

Figure 6 shows the Raman spectrum corresponding to the steel introduced into a 0.02 M solution of FeCl_2_ over a four-day period. It can be highlighted that the oxide film present at the steel surface after the exposition was colored in ocher. The main registered peaks that can be observed in Figure 4 appear at 249 cm^−1^, 374 cm^−1^, 521 cm^−1^, and 644 cm^−1^; thus, according to Faria [25], the identified compound for these peaks is lepidocrocite (γ-FeOOH).

**Figure 7:**
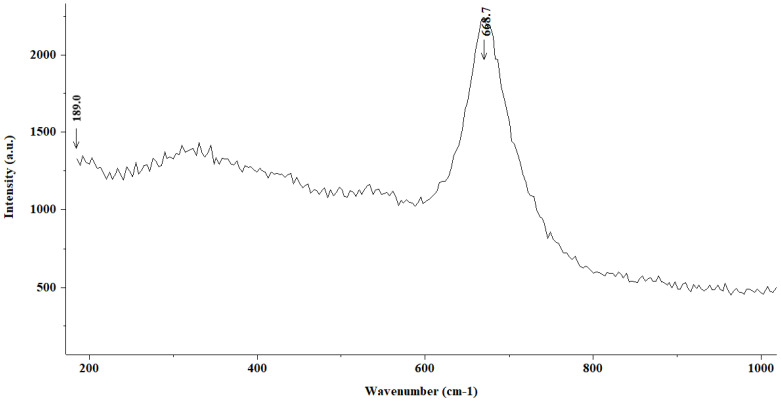
Raman spectrum (magnetite (Fe_3_O_4_)) corresponding to the steel introduced into a 0.02 M solution of FeCl_2_ on a black-colored area of the steel surface.

Figure 7 shows the Raman spectrum corresponding to the steel introduced into the same FeCl_2_ 0.02 M solution for a four-day period; however, in this case, the analysis was performed on an area of the steel surface colored in black. A broad band appears at 668 cm^−1^ that can be assigned to magnetite Fe_3_O_4_, of which the color is also black.

**Figure 8:**
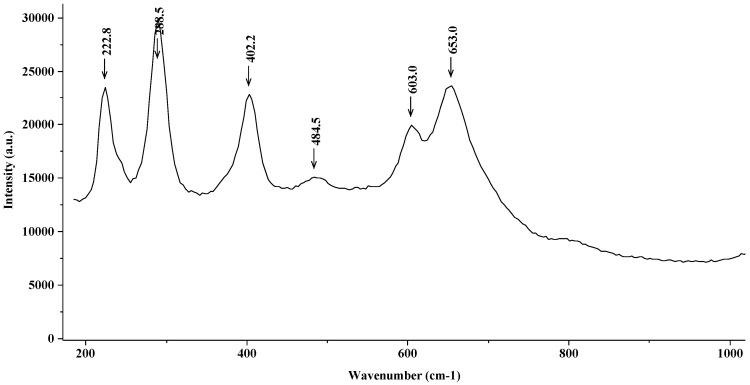
Raman spectrum (magnetite and hematite) corresponding to the steel introduced into a 0.2 M solution of FeCl_2._

Figure 8 shows the Raman spectrum corresponding to the steel in a FeCl_2_ 0.2 M solution. Again, the characteristic peaks corresponding to magnetite and hematite are observed.

**Figure 9:**
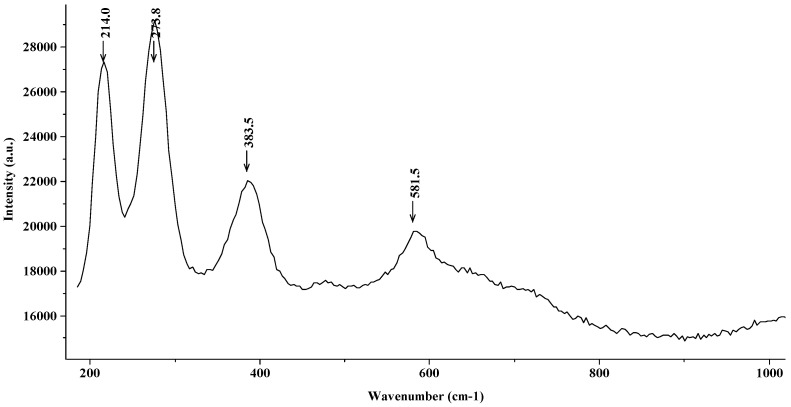
Raman spectrum (hematite) corresponding to the steel in a FeCl_2_ 2 M solution.

Figure 9 shows the Raman spectrum of the steel introduced in a FeCl_2_ 2 M solution. In this figure, corresponding to the most aggressive media, only the characteristic peaks of hematite (with a strong shift towards lower wavenumbers) are observed, with no appearance of magnetite, which has a lower oxidation state.

### 3.3. X-ray Photoelectronic Spectroscopy (XPS) Analyses

**Figure 10:**
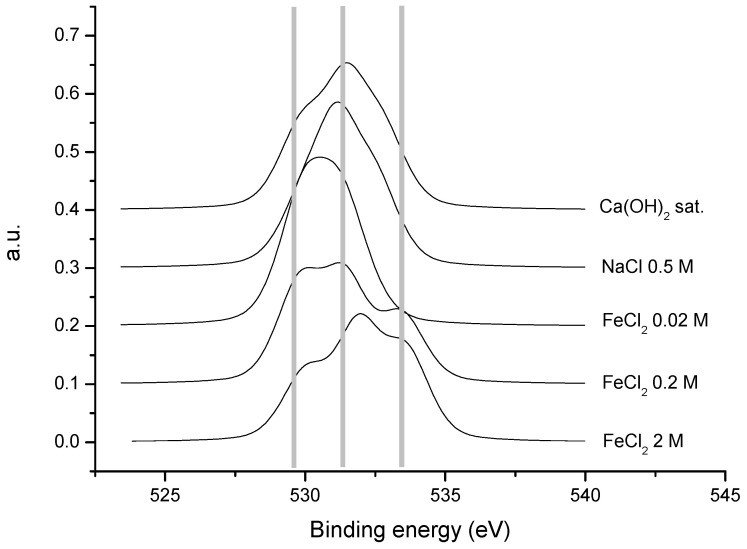
Normalized spectra of the transition oxygen 1 s for the five samples.

Figure 10 shows the normalized spectra of the transition oxygen 1 s for the five samples. For the solutions with FeCl_2_, a trend may be observed that shows an increase in the concentration of FeCl_2_. That is, an increase in the higher binding energies can be observed, as well as a general widening of the pulse, which is a consequence of the appearance of a greater number of chemical species in solution. The lowest binding energy (529.1 eV) corresponds to the oxygen forming part of one of the oxides, of Fe (II) or Fe (III), as described in the bibliography [1,2,27,28]. The highest values are assignable to oxygen in the hydroxyl group (532.2 eV) [3,5,16,29], reflecting the appearance of the oxyhydroxide of iron (III) and of the oxygen present in the molecule of H_2_O (533.0 eV) [7], which is present in the greatest quantities in the two samples with the highest concentration of FeCl_2_. This last contribution may be due to the lepidocrocite (γ-FeOOH), which may be partially hydrated [8,9,30,31].

**Figure 11:**
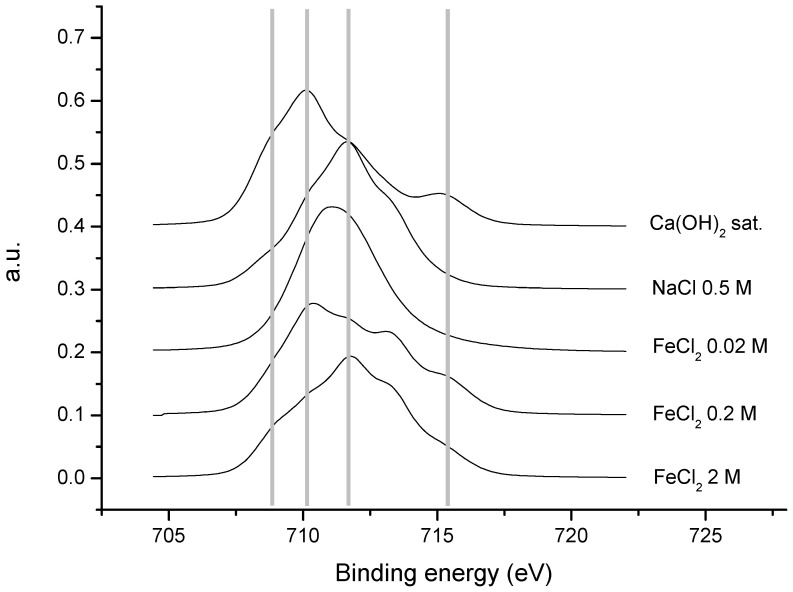
Normalized spectra of the transition of 2p_3/2_ of iron for the five samples.

The influence of the solution concentration and the length of treatment is confirmed by the examination of the spectra of the transition of 2p_3/2_ of iron (Figure 11). Upon increasing the concentration of FeCl_2_ in the solution, the peaks tend to shift to higher binding energy values, that is, iron in a higher oxidation state (Fe (III)), while in the mixed oxide, there is a combination of Fe^3+^ and Fe^2+^. In the sample treated with a solution of 0.2 M FeCl_2_, there are still contributions assignable to more reduced species (Fe (II) in the Fe_3_O_4_), whereas in the sample treated with 2 M FeCl_2_, these contributions are diminished, as one would expect from a sample coated with FeOOH. That is, one may interpret the evolution of Fe_3_O_4_ to a species of Fe(III), as indicated by the Raman spectroscopy results.

## 4. Discussion

The corrosion rates measured are an indication of the amount of oxides that formed in each solution. While the Ca(OH)_2_ solution only exhibited the passive film, which was too thin to be studied in detail by present techniques, the solution simulating sea water (0.5 M in NaCl) and that of 0.02 M of FeCl_2_ had similar corrosion rates. The more concentrated FeCl solutions showed a higher average corrosion rate (Figure 2).

These indications are reflected in the nature of the oxides. The first studies characterizing the most common oxidation products of iron were conducted by Thibeau et al. [13], Boucherit et al. [14], and Thierry [15]. They determined the characteristic frequencies of the Raman spectra for FeO, Fe_3_O_4_, α-Fe_2_O_3_, α-FeOOH, and γ-FeOOH. They were taken as the basis for analyzing the Raman spectra of the present work, although other authors have their own identification patterns [32,33,34,35]. Through the spectra, it was possible to notice that when no active corrosion was detected, the nature of the oxides shifted from maghemite to more oxidized and hydrated species (hematite, lepidocrocite, and magnetite), although their nature was common among the chloride-containing solutions. The only noticeable difference was that when black-color oxides were analyzed, only magnetite was found. In the other cases, hematite or lepidocrocite were the common species. The presence of akageneite was not detected in spite of the use of FeCl_2_ solutions, as was found by other researchers [35,36,37].

XPS techniques require a dry surface. To do that, the steel needs to be removed from the solution and paper-dried, which could modify the nature of the oxides. Using this technique, we proceeded to study the surface of the five samples. The information extracted from the spectra was only pertinent to the sample surface, as although the X-rays penetrated deep into the sample, the detected photoelectrons making up the spectra were emitted from the surface layers of the sample.

In the case of the steels treated with NaCl and Ca(OH)_2_, this last contribution was smoother. These spectra are quantified in Table 3, where the values and percentages of the binding energies and for the various contributions of oxygen 1 s are found for each sample. Effectively, in the samples containing FeCl_2_, a reduction in the percentage of oxygen assignable to an oxide was observed and a considerable increase in oxygen was identified with the OH groups. This is in agreement with the results obtained through the Raman spectroscopy, where with low concentrations of FeCl_2_, a layer of Fe_3_O_4_ appeared to form that evolved to γ–FeOOH with the increasing concentration of the FeCl_2_ solution. In the case of the steel sample in NaCl 0.5 M, the greatest portion of the oxygen is assignable to –OH, in concordance with the possible formation of γ-FeOOH, as detected in the Raman spectra.

Table 4 shows the values of the binding energies (eV) for the transition of 2p_3/2_ of iron. Five different contributions are evident [38,39], along with the contribution of each as a percentage of the total, so that one may better study the tendencies that experimental conditions exert on each of the samples.

The contributions assignable to the highest oxidation states (713.1 and 715.2 eV) grew when increasing the concentration of FeCl_2_. The least oxidized sample, Ca(OH)_2_, was the one with the highest percentage (almost 60% of the total) of the first two contributions (708.8 and 710.2 eV) that were related to the lowest oxidation states (Fe^2+^). The results obtained with XPS support the following interpretation: the formation of γ-FeOOH (lepidocrocite) for the samples was treated with FeCl_2_, while α-Fe_2_O_3_ (hematite) was detected in the 0.5 M NaCl solution and the presence of γ-Fe_2_O_3_ (maghemite) was found in the initial state before any corrosion in the Ca(OH)_2_ solution, according to Raman spectroscopy. It should be noted that lower binding energies arose from the oxygen of the oxide, but it was not possible to distinguish if they belonged to Fe^2+^ or Fe^3+^, since they were too close.

This would summarize the overall process as the following: in the saturated Ca(OH)_2_ solution, the oxides of the passive film were detected, mainly γ-Fe_2_O_3_ (maghemite); then, in the NaCl, the formation of new oxides, where some of them already contained Fe^2+^, were observed; and finally, as the FeCl_2_ concentration increased, a higher proportion of Fe^3+^ oxides was found with a high degree of hydration.

## 5. Conclusions

Although the results found in the present work are similar to those of other studies, they are not identical, confirming that the particular conditions of the test very much influence the nature and amount of the iron oxides that can be found during the corrosion of steel in the solutions simulating the concrete pore solution. The divergences indicate that the identification of the oxide types is not informative enough and that the electrochemical tests are better to characterize the corrosion stage. The features and species found in the present study were as follows:

- The corrosion rate of the steel was negligible in the solution of Ca(OH)_2_. However, the corrosion rate was progressively higher when increasing concentrations of FeCl_2_, while the solution of 0.5 M in NaCl presented values similar to the most diluted FeCl_2_ solution.
- The results obtained with Raman spectroscopy and XPS concerning the characterization of the progressive development of surface corrosion products during the period of propagation were mutually coherent. They showed that several types of iron oxides, dependent on the pH values, were detected.
- According to the techniques implemented in this research, which was focused on the evolution of the corrosion products of iron in a corrosion pit, it was confirmed that the higher the corrosion (i.e., the higher the amount of developed oxides), the higher the oxidation and hydroxylation.

## Figures and Tables

**Table 1:** Reinforcing steel composition.

% C = 0.30	% Si = 0.33	% Mn = 0.44	% S = 0.05
% P = 0.02	% Cr < 0.01	% Ni < 0.01	% Mo < 0.01

**Table 2:** Initial pH and conductivity of the solutions used as simulating the pit corrosion media.

Solution	pH	Conductivity (mS/cm)
Ca(OH)_2_ sat.	12.41	7.96
NaCl 0.5 M	6.55	45.5
FeCl_2_ 0.02 M	3.49	4.75
FeCl_2_ 0.2 M	2.65	31.9
FeCl_2_ 2 M	1.06	136.8

**Table 3:** Values and percentages of the binding energies and for the various contributions of the oxygen 1 s (*) binding energy (532 eV)_._

Binding Energy (eV)	FeCl_2_ 0.02 M	FeCl_2_ 0.2 M	FeCl_2_ 2 M	NaCl 0.5 M	Ca(OH)_2_ Sat.
529.9 ± 0.1	48.5	36.6	24.9	19.9	29.0
531.2 ± 0.5	37.7	38.4	42.8	52.0	45.5
533.0 ± 0.5	13.8 (*)	25.0	32.3	28.1	25.5

**Table 4:** Values and percentages of the binding energies and for the various contributions of 2p_3/2_ of iron.

Binding Energy (eV)	FeCl_2_ 0.02 M	FeCl_2_ 0.2 M	FeCl_2_ 2 M	NaCl 0.5 M	Ca(OH)_2_ (Sat.)
708.8 ± 0.1	7.1	11.2	12.7	7.6	21.2
710.2 ± 0.1	44.5	29.9	19.3	23.6	38.5
711.8 ± 0.1	25.2	22.9	33.4	41.8	20.2
713.1 ± 0.1	19.4	22.0	23.2	21.8	8.1
715.2 ± 0.1	3.8	13.9	11.4	5.2	12.0

## Data Availability

Not applicable.
